# In Vivo Light-Sheet Imaging of Senescence Reporter Activity in a Transparent Killifish

**DOI:** 10.21769/BioProtoc.5762

**Published:** 2026-07-20

**Authors:** Birgit Perner, Christoph Englert

**Affiliations:** 1 Molecular Genetics Lab, Leibniz Institute on Aging - Fritz Lipmann Institute (FLI), Jena, Germany; 2 Core Facility Imaging, Leibniz Institute on Aging - Fritz Lipmann Institute (FLI), Jena, Germany; 3 Institute of Biochemistry and Biophysics, Friedrich-Schiller-University Jena, Jena, Germany

**Keywords:** Light-sheet microscopy, Intravital imaging, *Nothobranchius furzeri*, Aging model, *cdkn1a (p21)* reporter, Transparent killifish, Cellular senescence, Fluorescence microscopy

## Abstract

Aging is associated with progressive accumulation of senescent cells, which contribute to tissue dysfunction and organismal decline. Conventional approaches for assessing cellular senescence, such as histological or immunofluorescence analyses of fixed tissue sections and flow cytometry, require tissue collection, thereby precluding longitudinal in vivo studies. To enable the analysis of cellular senescence in a living vertebrate model, we have previously generated a *cdkn1a (p21*)-driven GFP reporter line that was established in the transparent *klara* background of *Nothobranchius furzeri*. Here, we describe a protocol for in vivo light-sheet microscopy of the reporter activity as readout for senescence-associated cell cycle arrest with single-cell resolution. The procedure involves anesthesia and mounting of fish for stable positioning within the imaging chamber, with particular attention to animal welfare considerations. It further includes the acquisition of three-dimensional image stacks and subsequent image processing. The workflow allows monitoring of GFP-positive cells in intact living killifish at different developmental stages. Although imaging depth remains limited despite organismal transparency, this method provides high-resolution volumetric imaging with minimal phototoxicity and enables analysis of senescence dynamics in a short-lived vertebrate model. It is currently performed as a terminal procedure under approved ethical regulations, but longitudinal imaging would also be possible with additional ethical authorization.

Key features

• This protocol allows intravital imaging of fluorescent reporter activity in intact, anesthetized small teleost fish using light-sheet microscopy.

• Two complementary mounting strategies enable stable imaging across different developmental stages.

• A customized mounting support is rapidly prepared using UV-curable adhesive that fits directly into the standard Lightsheet Z.1 sample holder (Zeiss).

• Monitoring of vital parameters enables continuous assessment of the animal’s physiological state during imaging and early detection of stress or compromised well-being.

## Graphical overview


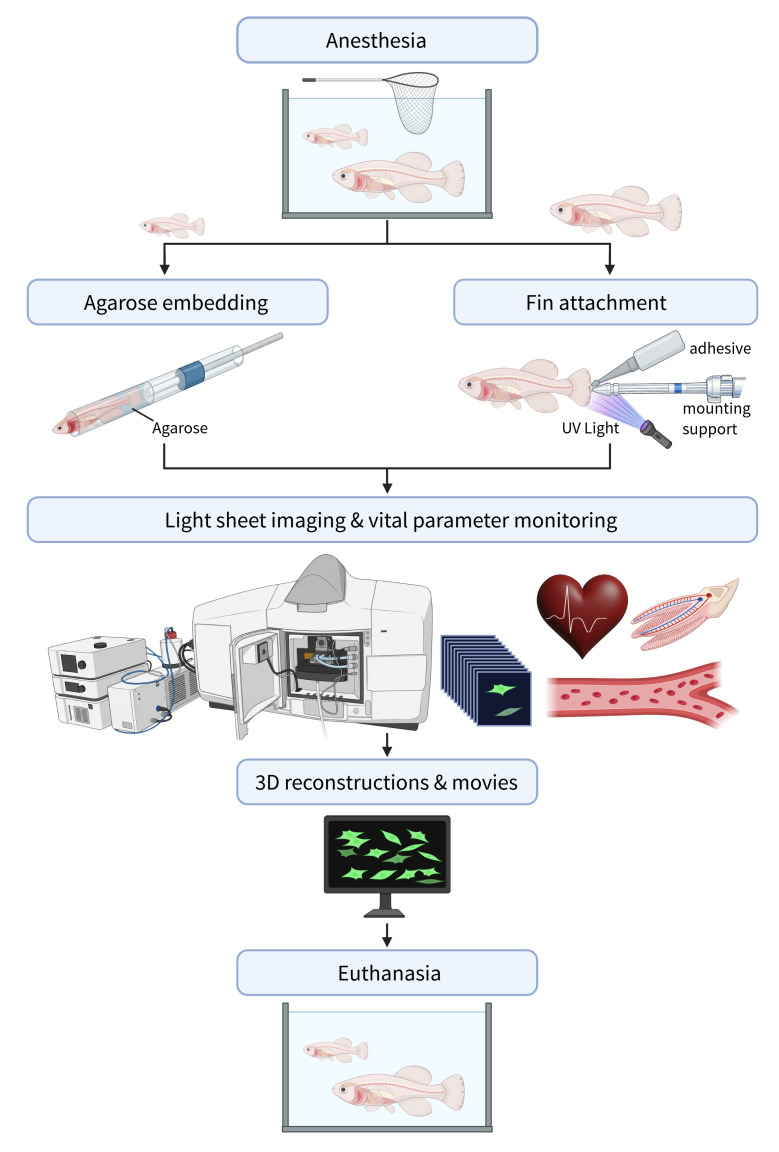


**Graphical overview of the workflow for intravital light-sheet imaging of small teleost fish**

## Background

Aging is associated with progressive accumulation of senescent cells across multiple tissues, which contribute to organ dysfunction and age-related pathologies, negatively influencing life span in vertebrates [1–5]. Experimental reduction of senescent cells in mice attenuated the functional decline of several organs [2], and pharmacological clearance using senolytic agents similarly improved tissue function and health span [4], highlighting the causal role of senescence in aging-associated deterioration. However, senescent cells can also exert beneficial functions, as transient senescence has been shown to promote wound healing and tissue repair [5,6]. Understanding the spatial and temporal dynamics of senescent cell accumulation is essential for dissecting their functional roles and for evaluating senescence-targeting interventions. The cyclin-dependent kinase inhibitor *cdkn1a (p21)* is a well-established mediator of cellular senescence and is commonly used as a marker to identify senescent cell populations in mammalian systems [1,3,6–8]. Notably, *cdkn1a (p21)* alone does not uniquely define cellular senescence, as its expression can also be induced in other contexts, such as during development, differentiation, or cell migration [9,10]; therefore, it should be interpreted in combination with other senescence features, including senescence-associated secretory phenotype (SASP), persistent cell cycle arrest, and characteristic morphological changes.

Beyond *p21*-based reporter systems, *p16 (Ink4a)*-based approaches, including luciferase and fluorescent knock-in models, represent another widely used strategy for studying senescence [2,11]. In addition, high-resolution imaging techniques, such as confocal and two-photon microscopy, have been employed to visualize senescence-associated processes. However, these approaches are often limited by tissue opacity and restricted imaging depth, which complicates whole-organism analyses.

Direct visualization of senescence dynamics in living vertebrates remains challenging due to limited light penetration into intact organisms and the restricted compatibility of conventional techniques with high-resolution volumetric imaging.

The African turquoise killifish *Nothobranchius furzeri*, characterized by a naturally short life span and the conservation of key aging hallmarks found in mammals, has emerged as a powerful vertebrate model for aging research [12–16]. To enhance the suitability of this model for imaging-based approaches, we previously developed the transparent killifish line, *klara* [17]. This line lacks melanophores, iridophores, and xanthophores, resulting in the absence of body pigmentation. Using this transparent platform, we generated a *cdkn1a (p21)*-driven GFP reporter line to monitor cellular senescence in vivo. In combination with light-sheet fluorescence microscopy, this approach enables rapid, minimally invasive three-dimensional visualization of *cdkn1a (p21)*-positive cells in living animals at high spatial resolution. We observed very few GFP-positive cells in 4-day-old fish, whereas their number increases markedly in older animals, indicating an age-dependent accumulation of *cdkn1a (p21)*-positive cells [17]. These results highlight the potential of this method for studying processes related to aging and regeneration in vivo.

Although light-sheet imaging is inherently constrained by light scattering and absorption within biological tissues [18], thereby limiting light penetration to superficial regions within the organism, the transparent background nevertheless facilitates intravital fluorescence imaging that would not be feasible in colored wild-type animals.

## Materials and reagents

**Biological materials**

1. Killifish reporter line (homozygous *M08-transparent-p21-ki-eGFP-NTR*) alias *p21 reporter;* generated in-house [17]

**Reagents**

1. Tricaine (3-ethoxycarbonylanilinium methanesulphonate, MS-222) (PHARMAQ AS, CAS number: 886-86-2)

2. Sodium bicarbonate (NaHCO_3_) (Roth, catalog number: 6885.1)

3. Low-melting-point agarose (Biozym, catalog number: 850081)

4. TopVision agarose tablets, normal melting point (Thermo Scientific, catalog number: 15225116)

**Solutions**

1. Tricaine solutions

a. Tricaine anesthetic solution (see Recipes)

b. Tricaine euthanasia solution (see Recipes)

2. 1% low-melting-point agarose (see Recipes)

3. 1.5% agarose, normal melting point (see Recipes)

**Recipes**

**1. Tricaine solutions**

Tricaine powder should be stored in a dry, light-protected environment at 4 °C to prevent degradation and the formation of potentially toxic byproducts. In accordance with institutional animal welfare regulations, the powder is supplied by the authorized veterinary unit in pre-aliquoted single-use tubes for either anesthesia or euthanasia, allowing accurate dosing and controlled handling. Sodium bicarbonate (NaHCO_3_) for buffering of tricaine solutions is likewise provided in pre-measured batches, ensuring standardized preparation of neutralized working solutions. This handling is further compliant with applicable European legislation [Regulation (EU) 2019/6] and relevant national regulations.

To prepare a 200 mL working solution, transfer 200 mL of system water (fish facility water) into a clean container and add the appropriate pre-aliquoted tubes of tricaine and NaHCO_3 _specified in the table below. Rinse each aliquot tube with a small volume of the prepared solution using a pipette to ensure complete transfer of the powder and mix gently until fully dissolved. Prepare the solution freshly on the day of application and allow it to equilibrate at room temperature.

**a. Tricaine anesthetic solution**

**Table:** 

Reagent	Final concentration	Quantity or volume
Tricaine	0.77 mM	40 mg
NaHCO_3_	4.76 mM	80 mg

**b. Tricaine euthanasia solution**

**Table:** 

solution	Final concentration	Quantity or Volume
Tricaine	3.85 mM	200 mg
NaHCO_3_	23.8 mM	400 mg

To prepare 200 mL of working solution, use the pre-aliquoted single-use euthanasia tubes following the same procedure described above for anesthesia.

**2. 1% low-melting-point agarose**

1% (w/v) low-melting-point agarose solution is prepared by dissolving 1 g of agarose in 100 mL of system water (fish facility). The mixture is heated using a microwave until the agarose is completely dissolved. Maintain at 37 °C to prevent solidification. Prepare the solution fresh on the day of the experiment.

**3. 1.5% agarose, normal melting point**

To prepare agar plates containing 1.5% (w/v) agarose, dissolve three agarose tablets in 100 mL of distilled water (according to the manufacturer’s instructions) and heat the mixture in a microwave until the agarose is completely dissolved. Pour the molten agarose into 90-mm Petri dishes (40 mL per dish) and allow to solidify at room temperature. Plates can be stored at 4 °C for several weeks if protected from drying and contamination.

**Laboratory supplies**

1. Lockable containers, 350 mL (Lock&Lock, catalog number: HPL806)

2. Safety container ROTILABO (Roth, catalog number: 8331.1)

3. Fish net (Pet shop)

4. Petri dishes, 90 mm (Greiner, catalog number: 628102)

5. Petri dishes, 60 mm (Greiner, catalog number: 632180)

6. Scalpel (Roth, catalog number: 1PYP.1)

7. Micro knife (FST, catalog number: 10316-14)

8. Glass capillaries designed as disposable glass caps for Transferpettor pipettes, volume range 100–200 µL (BRAND, catalog number: 701910)

9. Stainless steel capillary plungers with a Teflon tip designed as replacement piston rods for Transferpettor pipettes, volume 200 µL (BRAND, catalog number: 701938)

10. Luer-Lock syringe, 20 mL (Braun, catalog number: 4606205V)

11. Syringe extension (infusion tubing), 100 cm (Braun, catalog number: 40972629)

12. UV curable adhesive kit including applicator and a 395–505 nm UV light source with an intensity of approximately 60 mW/cm^2^ (e.g., Bondic Starter Kit, Bondic, https://bondic.shop/, catalog number: BONSTARTP1)

13. Fine-tipped sculpting tools (e.g., wax carving or modeling tools)

14. Pasteur pipettes, 4 mL, fine elongated tip (Roth, catalog number: 1CC9.1)

15. Pasteur pipettes, 6 mL, wide opening (Roth, catalog number: 1CCA.1)

16. Gel-loading tips, 0.5–10 µL (Hartenstein, catalog number: GS10)

17. High-precision tweezers, Dumont^®^ (Hartenstein, catalog number: PZ14)

18. Fine insect pins (Bioform, catalog number: B98-1)

19. Spring scissors (FST, https://finescience.com/, catalog number: 91500-09)

## Equipment

1. Light-sheet microscope (Zeiss, model: Lightsheet Z.1) equipped with dual-side illumination optics, temperature control modules (TempModule S1, TempModuleCZ-LSFM), PeltierBlock S, temperature sensor, sample chamber, and sample holder

*Note: For GFP imaging, samples were excited with a 488 nm laser using a quad-band laser blocking filter (LBF 405/488/561/640). Fluorescence was directed via a secondary beam splitter (SBS LP 560) through a bandpass emission filter (BP 505–545 nm) for detection. Imaging was performed using a 20× detection objective (W Plan-Apochromat, NA 1.0) and a sCMOS camera (pco.edge 4.2)*

2. Stereomicroscope (Zeiss, model: Stemi 305)

3. Thermomixer (Eppendorf)

4. Laboratory refrigerator (4 °C)

5. Timer or stopwatch

6. Imaging device for rapid documentation (e.g., smartphone with camera)

## Software and datasets

1. ZEN, black edition (Zeiss, release version 3.1; commercial software, license required), used for microscope control and image acquisition

2. ZEN, blue edition (Zeiss, release version 3.1; commercial software, license required), used for image processing and data export

3. 3Dxl rendering module (Zeiss, powered by arivis, integrated in ZEN 3.1 blue edition), used for three-dimensional reconstructions

*Notes: No free alternative is available for full functionality. However, ZEN Lite (Zeiss) provides a free version that supports basic visualization and three-dimensional reconstruction, although advanced processing steps such as dual-side fusion are not included. Open-source tools such as Fiji/Image J may also be used for basic image processing.*

## Procedure

**A. Preparation of the sample chamber and light-sheet microscope**

1. Assemble the imaging chamber according to the manufacturer’s instructions, including installation of the Peltier element for temperature control.

2. Insert the assembled chamber into the microscope and fill it with distilled water by using a 20 mL Luer-Lock syringe fitted with a syringe extension (infusion tubing). Carefully inspect the chamber for leakage.

3. If no leakage is detected, replace the water in the chamber with anesthetic solution. Ensure that the chamber is almost completely filled and free of air bubbles.

4. Start the microscope system and initialize the control software. Set the chamber temperature to 26 °C via the software-controlled Peltier unit and allow the system to equilibrate. This minimizes potential temperature fluctuations during imaging and ensures stable imaging conditions through continuous temperature regulation by the TempModule, Peltier Block, and temperature sensor.

*Notes:*

*1. Monitor the system for several minutes after filling the chamber with water, as minor leaks may only become apparent over time through small droplet formation at the chamber walls and a gradual decrease in water level.*

*2. The set temperature of 26 °C is optimal for the live fish sample. However, the system also supports a wide temperature range by heating or cooling the sample chamber. The TempModule LSFM serves as the dedicated control unit for the Peltier Block S, enabling rapid temperature adjustments between 10 and 42 °C within the sample chamber.*

**B. Anesthesia of fish**

*Note: Keep animals in the institutional fish facility under standard husbandry conditions until immediately before the experiment.*

1. Transfer the animals in a tank containing system water into a biosafety level 1 (BSL-1) approved transport container and carefully carry them to a BSL-1 microscopy room, while minimizing mechanical disturbances.

2. Gently transfer fish from the transport tank into a separate container filled with 200 mL of freshly prepared anesthetic solution using a fish net.

3. Verify successful anesthesia as indicated by the inability of the fish to maintain upright posture and the absence of a response to gentle tactile stimulation when touching the animal with a gel-loading pipette tip.

**Caution:** Do not discard anesthetic solution in the sink (see General note 7).

**Critical:** Only personnel trained and certified are authorized to anesthetize fish.

**C. Mounting and positioning of fish**

*Note: Select the mounting technique according to fish age and size: agarose embedding in capillaries is suitable for small fish that fit into the largest available capillary diameter (size 4, inner diameter ~2.15 mm, blue color code), where the widest part of the fish is slightly smaller than the inner capillary diameter to allow insertion without compression. Larger fish exceeding the capillary size should be mounted using the caudal fin attachment method. Specimens should not exceed the approximate dimensions of 12 mm in width and 20 mm in height, as also stated in the General notes section.*

**C1. Agarose embedding in capillaries**

1. Prepare an agar plate in advance (see Recipes) and allow it to equilibrate to room temperature.

2. Using a scalpel, cut a longitudinal groove into the surface of the agar plate. Shape one end of the groove into a gentle incline rather than an abrupt transition to the agar surface to facilitate controlled positioning of the fish.

3. Place a drop of anesthetic solution on the agar surface at the upper end of the inclined groove.

4. Transfer the anesthetized fish using a cut Pasteur pipette with a widened opening. Apply only gentle suction to minimize mechanical stress and prevent the animal from adhering to the plastic wall of the pipette. Position the fish such that the tail and trunk rest within the groove, while the head, including the opercular region, remains in the anesthetic droplet on the agar surface.

5. Carefully remove anesthetic solution from the groove using a fine Pasteur pipette and immediately replace it with 1% low-melting-point agarose at 37 °C. Always keep the head outside of the groove and avoid contact between the agarose and the gill region.

6. Using the appropriate plunger, first draw a small volume of low-melting-point agarose into the glass capillary. Position the capillary at the tail end of the fish and gently draw the agarose-embedded portion of the body into it. Gradually advance the capillary while continuing to draw the fish inward but keeping the head outside and in the anesthetic droplet on the agar surface.

7. Carefully reposition the capillary to allow the anesthetic-moistened head to be drawn inside the capillary without contacting the agarose.

8. Insert the capillary into the sample holder and allow the agarose to solidify.

9. Transfer the holder vertically into the temperature-controlled imaging chamber filled with anesthetic solution.

10. Gently press the plunger to extrude the agarose cylinder from the capillary until the embedded fish completely protrudes from the capillary.

**Critical:** Perform the entire embedding procedure rapidly and without interruption to prevent premature solidification of the low-melting-point agarose. Delays during capillary loading may lead to improper embedding and increased mechanical stress to the specimen. Keep the gill region free of agarose throughout the procedure to avoid impairment of opercular movement and subsequent respiratory distress. At the same time, prevent anesthetic solution from entering the agarose-filled groove, as dilution of agarose may impair proper solidification and result in insufficient mechanical stability to hold the specimen in place. Overall, the procedure is technically demanding and requires appropriate training or prior experience with capillary-based embedding techniques (see General note 6).

*Notes:*

*1. For optimal precision, the embedding procedure should be performed under a stereomicroscope. This facilitates accurate positioning of the fish within the agarose groove and helps ensure that the gill region remains free of agarose during capillary loading*.

*2. The orientation of the fish (lateral, dorsal, or ventral) during mounting does not critically affect imaging in our setup, as the Lightsheet Z1 is equipped with a rotatable sample holder that allows adjustment of the specimen orientation along its longitudinal axis prior to imaging. For setups without this feature, careful consideration of specimen orientation during mounting may be required.*

*3. For larger specimens, a modified agarose mounting approach using a truncated 1 mL syringe (Omnifi^®^-F, B. Braun, catalog number: 9161406V) can be employed in combination with an alternative sample holder system (Syringe Sample Holder Set, Zeiss, part number: 400100-8326-000). However, this setup is strictly limited by the inner diameter of the syringe barrel. Consequently, it is not suitable for specimens exceeding a maximum width of 4 mm, which precludes its use for 17-day post-hatching killifish, as their body diameter exceeds this threshold.*

**C2. Caudal fin attachment to a mounting support**

**Figure 1.**
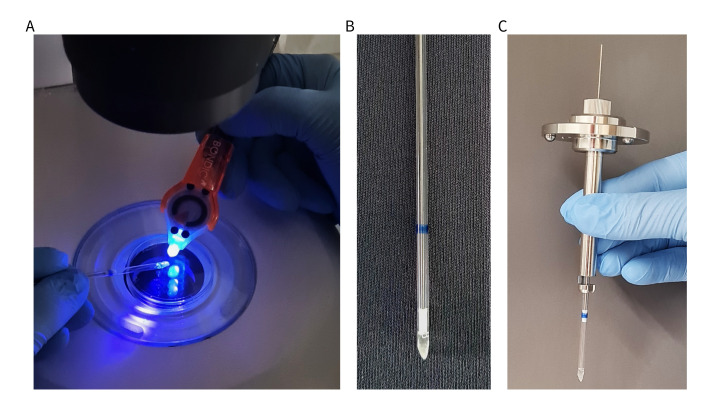
Preparation of customized mounting support for caudal fin attachment. (A) Modeling of a small platform at the distal end of a glass capillary using a UV-curable adhesive. (B) Fully cured mounting support with a triangular tip forming the attachment site for the caudal fin. (C) Modified capillary inserted into the standard sample holder of the light-sheet microscope.

**Figure 2.**
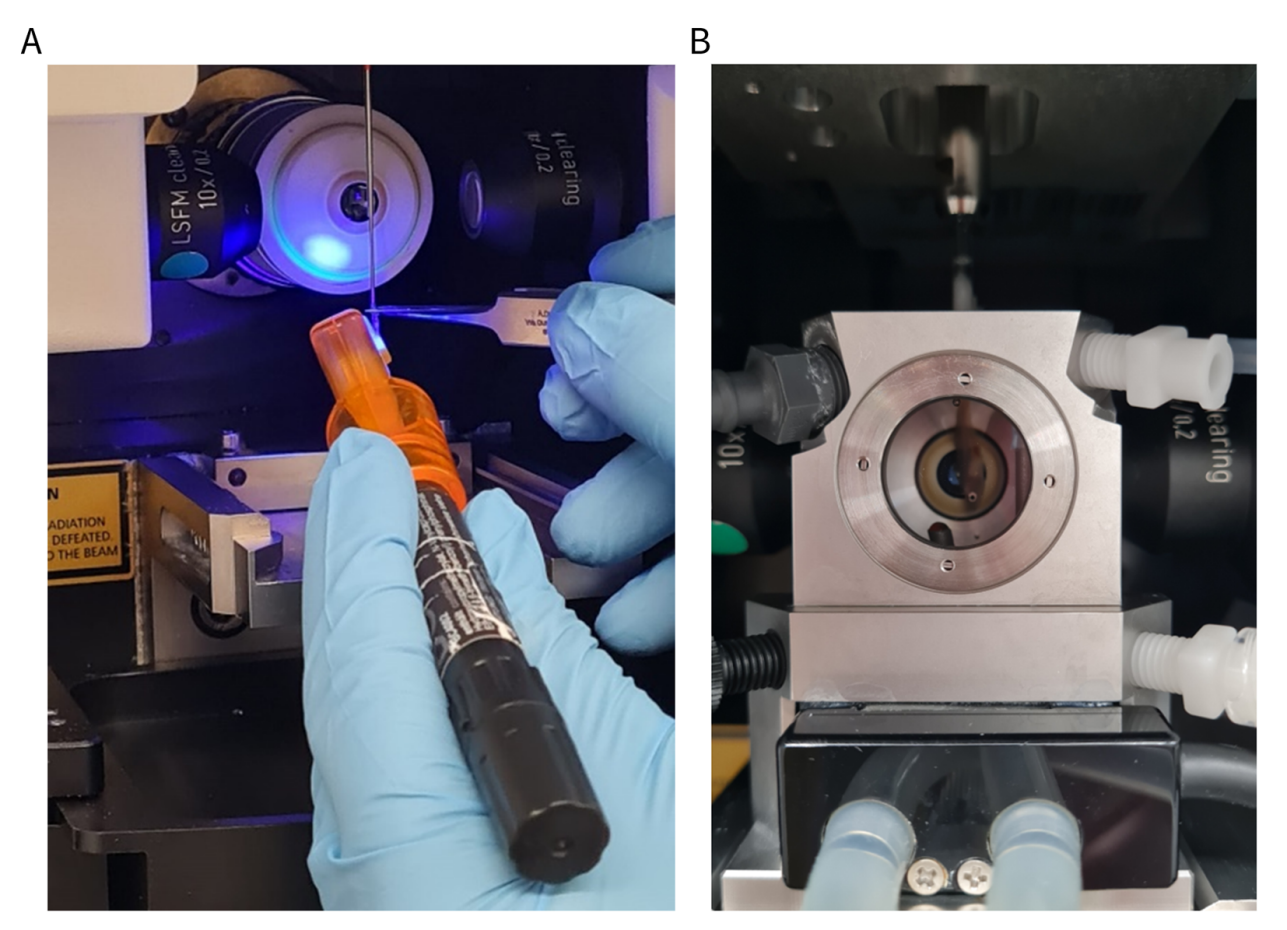
Mounting and positioning fish for light-sheet imaging by caudal fin attachment. (A) Attachment of the caudal fin tip to the customized mounting support using UV-curable adhesive while the support is positioned in a safe location within the microscope to avoid contact with the detection objective. (B) Mounted fish submerged in anesthetic solution within the sample chamber prior to imaging. The procedures shown here were approved by the local animal welfare authority and conducted in accordance with the German Animal Welfare Law and institutional guidelines.

1. To tailor a mounting support, use a UV-curable adhesive (Bondic UV repair system) to model a small platform (approximately 3 mm in diameter) at the distal end of a glass capillary. Apply the adhesive in small amounts and shape it using fine-tipped sculpting tools to create a flat, stable surface with the desired geometry for caudal fin attachment. Polymerize the adhesive using the UV light source provided with the Bondic system until complete curing is achieved (Figure 1A, B).

2. Using a 20 mL Luer-Lock syringe fitted with a syringe extension (infusion tubing), withdraw the prewarmed anesthetic solution from the sample chamber and set it aside for later use.

3. Remove the empty sample chamber from the microscope.

4. Insert the tailored mounting support into the sample holder (Figure 1C) and place the assembly into the microscope.

5. Using the microscope control software, move the sample holder with the mounting support downward and to a safe position along the z-axis, ensuring maximal distance from the detection objective to avoid accidental contact with the sample or contamination with adhesive during fin fixation.

6. Transfer the anesthetized fish to a container filled with anesthetic solution and place it close to the lowered mounting support.

7. Using Dumont forceps, grasp the caudal fin and attach a portion of it to the prepared platform. Apply a minimal amount of UV-curable adhesive to the fin tip, so that approximately 2–3 mm are fixed to the support. Expose the adhesive to UV light for several seconds until complete polymerization is achieved (typically 3–10 s; Figure 2A).

8. Use the microscope control software to raise the mounted specimen sufficiently to allow reinstallation of the sample chamber.

9. Reinsert the sample chamber into the microscope and refill it with the previously removed anesthetic solution.

10. Use the microscope control software to immediately lower the specimen into the chamber until it is fully submerged in the anesthetic solution (Figure 2B).

**Critical:** Fix only a small portion (2–3 mm) of the caudal fin to minimize tissue damage while ensuring stable positioning during imaging. Apply only a minimal amount of adhesive to avoid glue spreading and unintended adhesion of larger fin areas. The procedure should be performed quickly to minimize the risk of tissue drying during mounting. Prior practice of the procedure (e.g., by attaching fixed tissues or organs to various mounting supports) and assistance from a second person during mounting are strongly recommended (see General note 6).

*Notes:*

*1. This mounting approach is not suitable for very young fish. At early developmental stages, the small caudal fin may become substantially covered by the adhesive. Furthermore, due to buoyancy forces, the low body mass causes the specimen to bend upward and deflect away from the mounting support, moving it out of the imaging plane.*

*2. A commercially available Zeiss sample adapter (e.g., sample adapter short) can alternatively be used for specimen mounting. However, the modified capillary described here provides a customized attachment surface optimized for caudal fin fixation and fits directly into the standard sample holder supplied with the light-sheet microscope (eliminating the need for additional accessories).*

*3. Preselection of imaging orientation is recommended prior to mounting, as larger specimens may have limited rotational freedom within the imaging chamber due to spatial constraints (e.g., contact with chamber walls or the objective). GFP-positive cells were most reliably detected in a lateral orientation, particularly when imaging was focused on the fins.*

**D. Imaging**

1. Using the control software, position and orient the motorized sample holder, such that the specimen is optimally orientated for imaging of the region of interest.

2. Adjust the light sheet according to the manufacturer’s instructions.

3. Set laser power and exposure time to obtain sufficient fluorescent signal (here, GFP) while minimizing phototoxicity, using the lowest settings that still allow reliable detection of the fluorescent signal.

4. Acquire image stacks across the region of interest with an appropriate z-step size.

5. Continuously monitor vital parameters such as heartbeat, blood flow, and gill movements by frequently switching to the integrated overview camera between fluorescence acquisitions (Videos 1–4).

**Critical:** Perform imaging as quickly as possible and minimize setup time by determining the optimal orientation of the region of interest before mounting the animal when using the caudal fin attachment method. In larger fish, rotation of the specimen within the sample chamber becomes increasingly restricted, making preselection of the imaging plane and specimen orientation essential.

If vital parameters deteriorate during imaging (e.g., cessation of opercular movement or blood circulation), terminate the experiment immediately and humanely euthanize the animal as described in section E, following institutional animal welfare guidelines. In our experiments, imaging durations of approximately 10 min did not result in any observable deteriorations.

*Notes:*

**Figure 3.**
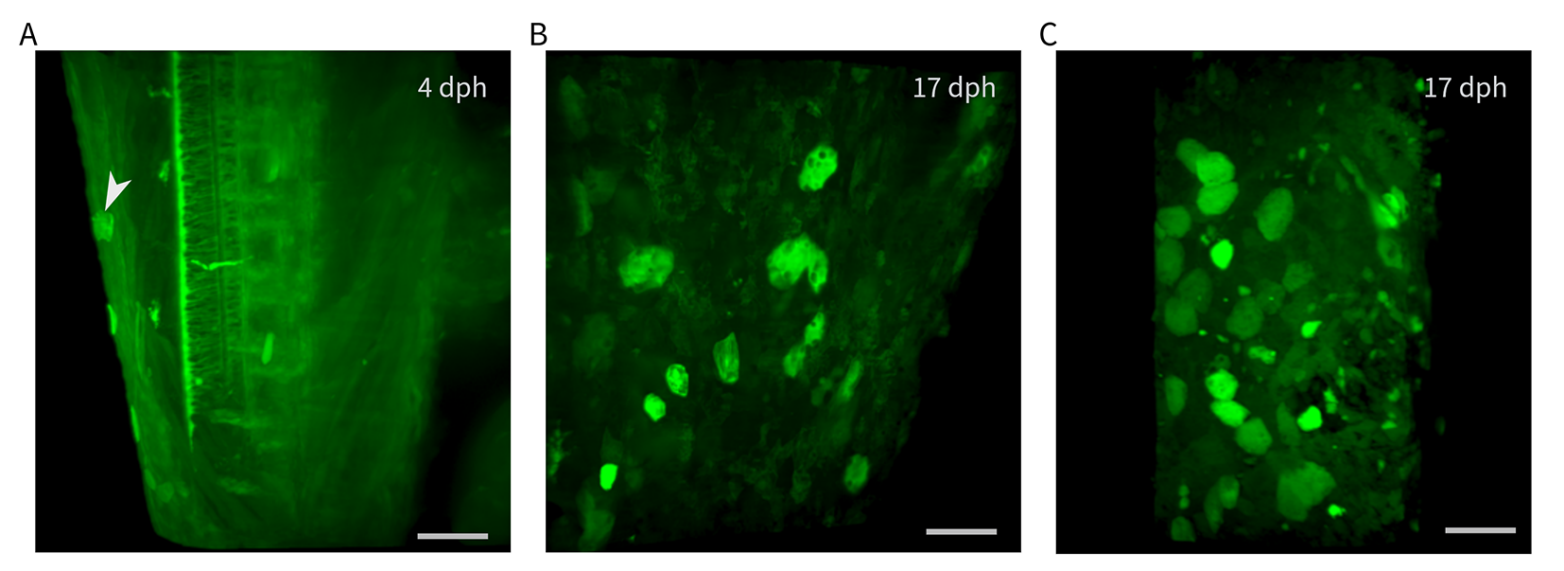
Detection of *cdkn1a (p21)-*positive cells in vivo at different ages. Representative three-dimensional reconstructions from light-sheet microscopy of *p21 reporter* fish show age-dependent accumulation of GFP-positive cells. (A, B) Comparison of dorsal fin regions at 4 days post-hatching (dph) and 17 dph. For consistency of the 3D reconstructions, a subset of 165 optical sections was extracted from the original dataset of 234 optical sections recorded for the 17 dph specimen. At 4 dph (A), very few GFP-positive cells are detectable, with a weakly fluorescent cell indicated by an arrowhead. (B) In contrast, numerous bright, enlarged GFP-positive cells are present at 17 dph. (C) A distinct region of a 17-dph fish (trunk) is shown, revealing abundant GFP-positive cells. This reconstruction is based on a z-stack of 380 optical sections. Scale bars, 100 μm. The experiments shown in this figure were approved by the local animal welfare authority and conducted in accordance with the German Animal Welfare Law and institutional guidelines.

*1. Imaging settings used in our experiments were as follows: 488 nm laser excitation at 20% laser power, exposure time of 100 ms, and a z-step size of 0.7 μm. Originally acquired image stacks used for Figure 3 consisted of 165 (Figure 3A), 234 (Figure 3B), and 380 (Figure 3C) optical sections. Stack size was selected depending on specimen age and the imaging depth achievable at the respective anatomical position. Imaging was performed using continuous drive acquisition, moving the sample continuously through the light sheet, enabling rapid z-stack acquisition. Under these imaging settings, no observable signs of photobleaching and phototoxicity were detected.*

*2. For documentation of vital functions, short video recordings can be captured from the microscope monitor using a smartphone camera while observing the specimen via the integrated overview camera (Videos 1–4).*

**Video 1.**
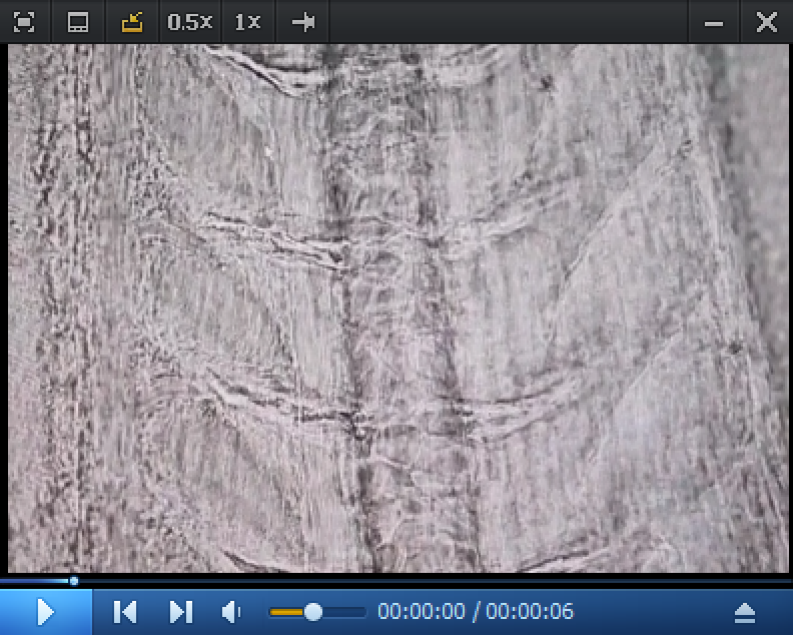
Blood flow in the trunk region of an anesthetized killifish (4 days post-hatching) during light-sheet microscopy. Circulating blood cells are visible in the trunk vasculature. Blood circulation represents one of the vital parameters monitored during live imaging. The specimen was mounted by agarose embedding in a glass capillary, positioned in the sample chamber of a light-sheet microscope (Lightsheet Z.1, Zeiss), and observed via the integrated overview camera (lateral view). The recording was captured from the system monitor using a smartphone camera. The experiment shown in this video was approved by the local animal welfare authority and conducted in accordance with the German Animal Welfare Law and institutional guidelines.

**Video 2.**
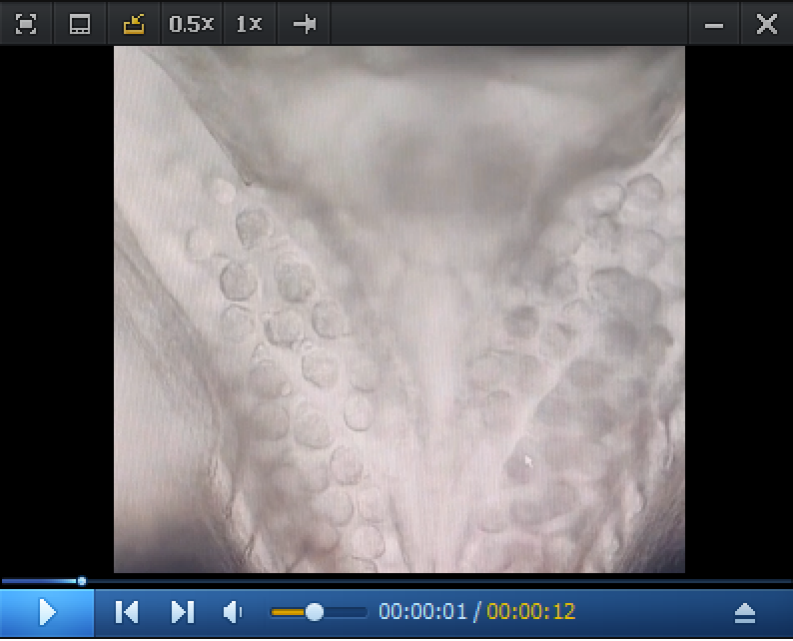
Cardiac activity of an anesthetized fish (4 days post-hatching) during light-sheet microscopy. The beating heart is clearly visible from the dorsal perspective. Cardiac activity represents one of the vital parameters monitored during live imaging. The specimen was mounted by agarose embedding in a glass capillary, positioned in the sample chamber of a light-sheet microscope (Lightsheet Z.1, Zeiss), and observed via the integrated overview camera. The recording was captured from the system monitor using a smartphone camera. The experiment shown in this video was approved by the local animal welfare authority and conducted in accordance with the German Animal Welfare Law and institutional guidelines.

**Video 3.**
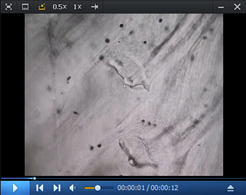
Blood flow of an anesthetized fish (17 days post-hatching) during light-sheet microscopy. Circulating blood cells are visible in vessels at the base of a fin. Blood circulation represents one of the vital parameters monitored during live imaging. The specimen was mounted by caudal fin attachment to a mounting support, positioned in the sample chamber of a light-sheet microscope (Lightsheet Z.1, Zeiss), and observed via the integrated overview camera (lateral view). The recording was captured from the system monitor using a smartphone camera. The experiment shown in this video was approved by the local animal welfare authority and conducted in accordance with the German Animal Welfare Law and institutional guidelines.

**Video 4.**
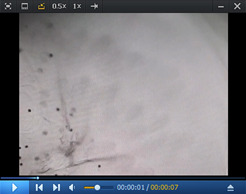
Gill movement of an anesthetized fish (17 days post-hatching) during light-sheet microscopy. Rhythmic gill movement indicating respiration is visible and represents one of the vital parameters monitored during live imaging. The specimen was mounted by caudal fin attachment to a mounting support, positioned in the sample chamber of a light-sheet microscope (Lightsheet Z.1, Zeiss), and observed via the integrated overview camera (lateral view). The recording was captured from the system monitor using a smartphone camera. The experiment shown in this video was approved by the local animal welfare authority and conducted in accordance with the German Animal Welfare Law and institutional guidelines.

**E. Post-imaging euthanasia**

At the end of the imaging experiment, fish are euthanized by immersion in a euthanasia bath. The procedure depends on the mounting method used.

**E1. Fish embedded in agarose within a capillary**

1. Using the plunger, gently extrude the part of the agarose cylinder with the embedded fish from the capillary into a small Petri dish.

2. Keep the specimen moist with anesthetic solution while gently extracting it from the agarose using fine insect pins, preferably under a stereomicroscope to ensure precise handling.

3. Transfer the fish into a large container filled with 200 mL of euthanasia solution (euthanasia bath).

4. Set a timer for 5 min and confirm death (as described in section E3).

**E2. Fish mounted via caudal fin attachment**

1. Using spring scissors, cut the caudal fin just below the adhesive attachment to release the fish from the mounting support and allow it to fall into a small, wet fish net.

2. Immediately transfer it into a container filled with 200 mL of euthanasia solution (euthanasia bath).

3. Set a timer for 5 min and confirm death (as described in section E3).

**E3. Confirmation of death**

1. Confirm death based on the following criteria: absence of body movement, absence of opercular movement, absence of startle response, and no response after gentle pinching of the caudal fin.

**Caution:** Do not discard euthanasia solution in the sink (see General note 7).

**Critical:** Only personnel who are trained and certified are authorized to euthanize fish.

2. If any of the above-mentioned criteria are not met, extend immersion in euthanasia solution for an additional 5 min and reassess.

*Note: Although fish can be released from agarose after imaging without apparent harm, the minor tissue damage caused by caudal fin attachment is expected to regenerate rapidly, and imaging using light-sheet microscopy is generally fast and gentle, animals were nevertheless euthanized at the end of the experiment to prevent any potential pain, distress, or suffering. Death is confirmed only when all criteria are met.*

**F. Image processing**

1. Process image stacks using ZEN software (blue edition, Zeiss). Perform dual side fusion if required and adjust brightness and contrast.

2. Generate three-dimensional reconstructions of the recorded z-stacks using the 3Dxl rendering module (powered by arivis) implemented in ZEN and export the processed image data as TIFF files using the export function provided by ZEN (Figure 3A–C).

3. Alternatively, employ the 3D module to create an animated visualization of the three-dimensional reconstructions and export them as movies in AVI format using the corresponding export function in ZEN (Video 5).

*Note: To ensure consistency of 3D reconstructions and enable comparison between developmental stages, a defined subset of optical sections can be extracted from the original image stack using the corresponding tool in the ZEN software. This approach was used to select 165 optical sections from a dataset of 234 sections acquired from a 17 dph specimen (Figure 3B and Video 6).*

**Video 5.**
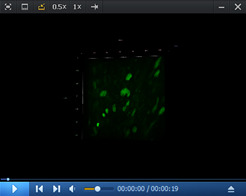
Animation of a three-dimensional reconstruction of GFP-positive cells in the dorsal fin of an anesthetized *p21 reporter* fish at 17 days post-hatching mounted by caudal fin attachment. The 3D rendering of 234 optical sections reveals numerous bright, enlarged cells and illustrates their morphology and spatial distribution within the tissue context. The specimen was positioned in the sample chamber of a light-sheet microscope (Lightsheet Z.1, Zeiss). The animation was generated using the ZEN software (blue edition, Zeiss). The experiments shown in this video were approved by the local animal welfare authority and conducted in accordance with the German Animal Welfare Law and institutional guidelines.

**Video 6.**
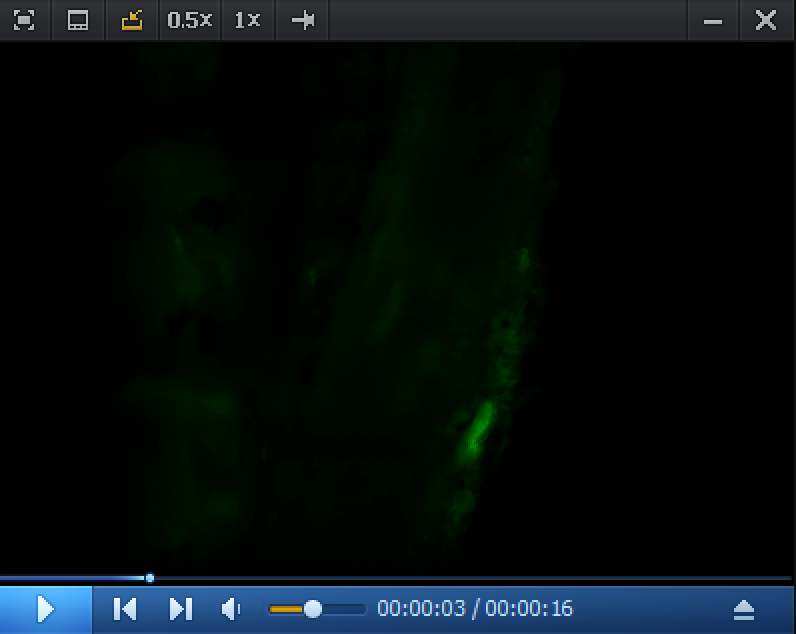
Unprocessed z-stack of optical sections recorded from the dorsal fin of an anesthetized *p21 reporter* fish at 17 days post-hatching, mounted via caudal fin attachment. The specimen was positioned in the sample chamber of a light-sheet microscope (Lightsheet Z.1, Zeiss). The dataset consists of 165 optical sections acquired at 488 nm (20% laser power), 100 ms exposure time, and a z-step size of 0.7 μm. The movie displays the image stack used for 3D projection in Figure 3B. Movie export was performed using the ZEN software (blue edition, Zeiss) and is displayed at 10 frames per second. The experiments shown in this video were approved by the local animal welfare authority and conducted in accordance with the German Animal Welfare Law and institutional guidelines.

## Validation of protocol

This protocol has been used and validated in the following research article: Krug et al. [17]. Generation of a transparent killifish line through multiplex CRISPR/Cas9mediated gene inactivation. eLife (Figure 5J–M). In this study, light-sheet microscopy was applied to visualize *cdkn1a (p21)* reporter activity in intact killifish and to assess stage-dependent changes in the occurrence and accumulation of GFP-positive cells during development. Analyses were performed on multiple independently imaged animals (n = 7 per developmental stage) to ensure reproducibility of the observed GFP signal patterns. Consistent stage-dependent differences in the number of detectable reporter-positive cells between animals at 4 days post-hatching and 17 days post-hatching were observed across all analyzed specimens. Representative images are shown in [17] and in Figure 3 of this manuscript. Sex was not assessed, as experiments were conducted at developmental stages prior to reliably distinguishable morphological sexual differentiation in killifish.

## General notes and troubleshooting

**General notes**

1. This protocol is optimized for live imaging using the Lightsheet Z.1 microscope (Zeiss). When applying the procedure to other light-sheet microscopy systems, certain steps, particularly those related to sample mounting, require adaptation according to the specifications of the respective instrument.

2. As low-melting-point agarose has optical properties and refractive index similar to those of the surrounding aqueous medium, no major differences in image quality are expected solely as a result of the mounting method used. Both approaches enable the qualitative assessment of the presence and distribution of reporter-positive cells rather than quantitative fluorescence intensity measurements. Consequently, potential minor optical differences associated with the mounting method are not expected to affect the biological conclusions drawn from the imaging data.

3. The customized mounting strategies described here allow the insertion of a specimen up to approximately 12 mm in width and 20 mm in height into the Lightsheet Z.1 sample chamber. The accessible imaging volume may be smaller depending on the mounting configuration and specimen geometry.

4. In addition to geometric constraints, increasing specimen size leads to stronger light scattering and absorption within the tissue, which causes reduced image quality and limits the effective imaging depth. This should be considered when selecting developmental stages or experimental models for live imaging.

5. The entire procedure constitutes an animal experiment and must be approved in detail by the appropriate authority before initiation.

6. Minimize the duration of each animal’s participation in the procedure. In our hands, the time from anesthetizing the fish to final euthanasia does not exceed 25 min per animal. Representative timing estimates are as follows: mounting to acquisition of the first image stack typically takes 5–9 min, and completion of the imaging stack to transfer the animal into euthanasia solution requires less than 1 min. Similar time requirements were observed for both mounting approaches, as the longer agarose embedding procedure is offset by the additional chamber filling step required for caudal fin attachment. Depending on the exact time required for mounting, up to six image stacks were acquired per individual fish within a single imaging session.

7. The method involves delicate manual handling and technical operation of the imaging system and therefore benefits from prior experience and training. Practicing the procedure using fixed specimens or tissues is recommended to develop the skills necessary for reliable and reproducible execution.

8. Collect all tricaine-containing solutions separately in a designated, sealable waste container and dispose of them according to institutional regulations. Tricaine-containing solutions must never be poured down the sink.

**Troubleshooting**

**Problem 1:** Presence of air bubbles in the sample chamber.

Possible cause: Air was introduced during filling of the chamber or during introduction of the sample.

Solution: Carefully and promptly refill the chamber with anesthetic solution while avoiding the introduction of air bubbles. Keeping a syringe prefilled with anesthetic solution readily available facilitates rapid refilling and helps prevent the introduction of additional air bubbles caused by repeated aspiration and refilling. Remove any bubbles before starting image acquisition.

**Problem 2:** Premature agarose solidification during embedding.

Possible cause: Delayed handling.

Solution: Re-expose the specimen to the anesthetic solution to maintain immobilization. Carefully release the animal from the partially solidified agarose using two insect pins and re-embed the specimen following the described embedding procedure (steps C1.3–7).

**Problem 3:** Deterioration of physiological parameters during or immediately after mounting.

Possible cause: Mechanical stress during mounting.

Solution: Check for heart activity and opercular movement. If observed, terminate the experiment immediately and process the animal according to the Critical notes in section D.

**Problem 4:** Insufficient immobilization resulting in specimen drift during image acquisition.

Possible causes: Incomplete anesthesia, incomplete agarose solidification, or suboptimal fixation of the caudal fin to the mounting support.

Solutions:

For agarose-embedded specimen: Remove the specimen from the mounting setup and return it to the anesthetic solution. Use two insect pins to retrieve the agarose-embedded specimen and repeat the procedure described in steps C1.3–10.

For specimens mounted via caudal fin attachment: Carefully improve fixation by applying additional adhesive to stabilize the position on the mounting support.

In both cases, do not exceed a total duration of 25 min per specimen for the experimental procedure, as specified in the General notes section.

**Problem 5:** Weak fluorescence signal.

Possible causes: Laser power or exposure time too low, specimen not optimally positioned within the light sheet.

Solutions: Increase laser power or exposure time carefully. Reposition the specimen so that the region of interest is properly aligned with the light sheet.

**Problem 6:** Uneven illumination or shadowing artifacts.

Possible causes: Misalignment of the light sheet relative to the specimen or obstruction within the optical path.

Solution: Readjust the light sheet according to the manufacturer’s instructions and ensure that the specimen is properly positioned.

**Problem 7:** Reduced image quality at greater imaging depth.

Possible cause: Increased light scattering and absorption in thicker tissue.

Solutions: Reduce imaging depth where possible or select smaller animals (earlier developmental stages).
